# Serum Levels of VEGF-A and Its Receptors in Patients in Different Phases of Hemorrhagic and Ischemic Strokes

**DOI:** 10.3390/cimb44100332

**Published:** 2022-10-14

**Authors:** Anastasiya S. Babkina, Mikhail Ya. Yadgarov, Irina V. Ostrova, Vladislav E. Zakharchenko, Artem N. Kuzovlev, Andrey V. Grechko, Maxim A. Lyubomudrov, Arkady M. Golubev

**Affiliations:** Federal Research and Clinical Center of Intensive Care Medicine and Rehabilitology, 107031 Moscow, Russia

**Keywords:** ischemic stroke, hemorrhagic stroke, brain damage, vascular endothelial growth factor, VEGFR-1, VEGFR-2

## Abstract

Vascular endothelial growth factors (VEGFs) are important regulators of angiogenesis, neuroprotection, and neurogenesis. Studies have indicated the association of VEGF dysregulation with the development of neurodegenerative and cerebrovascular diseases. We studied the changes in serum levels of VEGF-A, VEGFR-1, and VEGFR-2 in patients at various phases of ischemic and hemorrhagic strokes. Quantitative assessment of VEGF-A, VEGFR-1, and VEGFR-2 in serum of patients with hemorrhagic or ischemic stroke was performed by enzyme immunoassay in the hyper-acute (1–24 h from the onset), acute (up to 1–7 days), and early subacute (7 days to 3 months) phases of stroke, and then compared with the control group and each other. Results of our retrospective study demonstrated different levels of VEGF-A and its receptors at various phases of ischemic and hemorrhagic strokes. In ischemic stroke, increased VEGFR-2 level was found in the hyper-acute (*p* = 0.045) and acute phases (*p* = 0.024), while elevated VEGF-A and reduced VEGFR-1 levels were revealed in the early subacute phase (*p* = 0.048 and *p* = 0.012, respectively). In hemorrhagic stroke, no significant changes in levels of VEGF-A and its receptors were identified in the hyper-acute phase. In the acute and early subacute phases there was an increase in levels of VEGF-A (*p* < 0.001 and *p* = 0.006, respectively) and VEGFR-2 (*p* < 0.001 and *p* = 0.012, respectively). Serum levels of VEGF-A and its receptors in patients with hemorrhagic and ischemic stroke indicate different pathogenic pathways depending on the phase of the disease.

## 1. Introduction

Acute cerebrovascular accidents have major medical and social significance. Due to the high global mortality and morbidity of stroke, improved differential diagnosis of its types, disease control methods at different phases, and outcome prediction are essential. The pathways to vascular endothelial growth factor (VEGF) secretion are different for various types of stroke. Ischemic stroke causes reduction of tissue oxygenation, resulting in energy deficit and subsequent neuronal death. If the blood flow interruption is incomplete, a penumbra zone appears, where the damaged cells remain in a necrotic-like condition, and an increase in excitotoxicity occurs, causing destruction of cytosolic structures. Ischemia also affects vessels and stimulates VEGF secretion [1]. Subarachnoid hemorrhages (SAH) usually develop as a result of either arterial aneurysm rupture or traumatic brain injury. Vascular wall damage induces VEGF production. In some cases, SAH can be caused by anticoagulant medications, blood coagulation disorders, etc. The spread of blood into the cerebrospinal fluid space can result in intracerebral hypertension, blockage of the cerebrospinal tracts by blood clots, and cellular response associated with lysis. Intracerebral hemorrhage is caused by a rupture of the altered brain vessels (commonly in hypertension), or red blood cell diapedesis. The main pathogenetic factor here is arterial hypertension. In ischemic stroke, damage to microcirculatory blood-vessel walls occurs, leading to the development of hemorrhages with diapedesis. Abnormalities of coagulation and fibrinolysis along with vascular ischemia also play important roles in triggering VEGF production [2].

Studying levels of molecular markers of brain damage and regeneration depending on the type and phase of stroke is a promising area of research [3,4,5,6].

Several clinical studies have reported increased serum levels of vascular endothelial growth factor (VEGF) in patients with stroke of various etiologies and severity [7,8,9,10].

Angiogenesis processes mediated by vascular growth factor receptors are known to start within a few hours after stroke onset [11]. Vascular endothelial growth factor (VEGF), also known as vascular permeability factor (VPF), is involved in hypoxia-induced neovascularization by specifically binding to vascular endothelial cells and thus promoting their growth [12,13].

Levels of circulating VEGF usually are very low in healthy subjects, and are important for maintaining endothelial viability and basic transport across the endothelial barrier. The main storage sites of circulating VEGF are α2-macroglobulin, sVEGFR-1 (sFlt-1), and platelets, which release VEGF at activation in vivo or in vitro [14]. The levels of circulating VEGF increase in response to hypoxia, inflammation, and immunopathological processes. A significant difference was found between VEGF levels in Alzheimer’s disease patients and healthy individuals [15]. A number of studies have shown the diagnostic and prognostic value of circulating VEGF in arterial hypertension [16], coronary artery disease [17], myocardial infarction [18], peripheral arterial disease, and heart failure [19,20].

VEGF-A isoform is the most potent angiogenic factor within the VEGF molecule family.

The biological activity of VEGF-A is mediated by two receptors, VEGFR-1 and VEGFR-2 [20]. Despite the stronger affinity of VEGF-A to VEGFR-1, signals regulating proliferation, survival, endothelial cell migration, and changes in vascular permeability are predominantly transmitted through VEGFR-2, which has the strongest tyrosine kinase activity [21]. It has been noted that the production of VEGFR-1 and VEGFR-2 increases in response to hypoxia, but less so than VEGF [20]. The exact mechanism of action of VEGFR-1 is not fully understood. However, it is known that the angiogenic response to hypoxic conditions is induced by the binding of VEGF-A to VEGFR-2, which stimulates the growth of vascular endothelial cells. Soluble VEGFR-1 (sFlt-1) is a circulating form of VEGFR-1 with high affinity for VEGF. Acting as a decoy, sFlt-1 (also regulated by hypoxia) binds circulating VEGF, inhibiting the angiogenic action of VEGF binding to endothelial cell-membrane-bound VEGFR-2. Consequently, sFlt-1 is a negative regulator of angiogenesis [22]. An experimental study by Cárdenas-Rivera et al. showed that endogenous VEGFR-2 activation interferes with neuroprotective mechanisms mediated by VEGFR-1 activation [23].

VEGF-A and its receptors are expressed predominantly in endothelial cells, as well as in hematopoietic cells, neutrophils, smooth muscle cells, retinal progenitor cells, and tumor cells, according to various studies [24,25,26,27,28]. The distribution of receptors in the blood–brain barrier vessels is not uniform, with VEGF-1 prevailing on the luminal and VEGF-2 on the abluminal side of the endothelium. Activation of VEGFR-1 on the luminal side results in a cytoprotective response, whereas activation of VEGFR-2 induces vascular permeability [29].

There have been few studies showing weak expression of VEGF-A (astrocytes), VEGFR-2 (neurons), and VEGFR-1 (pericytes) in the cells of the central nervous system [30,31]. Studies on cell cultures have shown that under oxygen and nutrient deficiency, neurons showed increased expression of VEGFR-2, VEGFR-1, and VEGF [32].

Vascular endothelial growth factor and its receptors, initially found in the vascular system, were long believed to be involved only in angiogenesis. The mechanisms of impact of the VEGF/VEGFR signaling system on the proliferation, migration, apoptosis, and permeability of endothelial cells in normal conditions and under hypoxia, including in cancer and circulatory disorders, have been well studied [30,33].

However, studies of the role of vascular endothelial growth factor in the central nervous system revealed a direct effect of the VEGF/VEGFR signaling system on neuronal progenitor cells, their differentiation, neuronal migration, neuronal survival, and recovery after injury [34,35]. VEGFs are important regulators of angiogenesis, neuroprotection, and neurogenesis, which has been confirmed by studies indicating the association of VEGF dysregulation with the development of neurodegenerative and cerebrovascular diseases [35]. There is evidence of a direct link between blood VEGF levels and the extent of brain damage [36,37,38]. Most of the studies on this subject have been experimental. Few clinical studies have revealed increased serum VEGF-A levels in acute cerebrovascular accidents. However, they have tended not to consider biomarker changes in the blood of patients with various phases of strokes and have separately assessed serum VEGF-A levels in patients, rather than in combination with its receptor levels.

Therefore, a study of serum levels of VEGF-A and its receptors during various phases of ischemic and hemorrhagic stroke is particularly relevant.

Objective: to characterize the changes in serum levels of VEGF-A, VEGFR-1, and VEGFR-2 in patients at various phases of ischemic and hemorrhagic strokes.

## 2. Materials and Methods

### 2.1. Study Subjects

The retrospective cohort study included patients with ischemic and hemorrhagic stroke hospitalized in the intensive care units of the Federal Research and Clinical Center of Intensive Care Medicine and Rehabilitology and the M. P. Konchalovsky City Clinical Hospital No. 3 in Moscow, Russian Federation (inclusion period: 1 January 2018–1 November 2019). The control group consisted of 40 volunteers (apparently healthy people) (Table S1). Informed consent was obtained from the volunteers before inclusion in the study.

The study design was based on the STROBE recommendations for observational studies (cohort studies and case–control studies) [39].

Inclusion criteria were clinical signs of stroke as confirmed by computed brain tomography, any localization of stroke, and level of consciousness on admission at 4–9 points on the Glasgow Coma Scale. There were no age or comorbidity limitations.

Exclusion criteria were unstable hemodynamic parameters during the hyper-acute phase of stroke; consciousness level below 4 on the Glasgow Coma Scale, infectious complications, sepsis, and terminal condition.

The diagnosis of stroke was made according to the guidelines of the Ministry of Health of the Russian Federation (2020).

The study protocol was approved by the local bioethics committee of the Federal Research and Clinical Center of Intensive Care Medicine and Rehabilitology (protocol 4/21/3 from 21 September 2021). Data were collected and analyzed independently by two investigators using a medical information system and paper medical records.

The primary end point of the study was measurement of the levels of VEGF-A, VEGFR-1, and VEGFR-2 in different phases of ischemic and hemorrhagic stroke. Quantitative assessment of VEGF-A, VEGFR-1, and VEGFR-2 in serum of patients with hemorrhagic or ischemic stroke was performed by enzyme immunoassay in the hyper-acute (1–24 h from the onset), acute (up to 1–7 days), and early subacute (7 days to 3 months) phases of stroke. We used the stroke timeline framework proposed by the Stroke Recovery and Rehabilitation Roundtable Taskforce [40].

### 2.2. Blood Samples and ELISA Technique

Blood samples of 8 mL were taken from the antecubital vein on an empty stomach, from patients with hyper-acute, acute, and early subacute stroke, as well as from healthy volunteers. Blood samples were stored in standard tubes with EDTA at room temperature for 0.5 h. Blood samples were then centrifuged at 2000 rpm for 10 min to separate the serum. Afterwards, the samples were immediately placed in 0.25 mL Eppendorf tubes at −20 °C.

The serum levels of VEGF-A, VEGFR-1, and VEGFR-2 were quantified using Human VEGF-A Platinum ELISA (Thermo Fisher Scientific, Vienna, Austria), ELISA Kit for Vascular Endothelial Growth Factor Receptor 1 (VEGFR1) (Cloud-Clone Corp., Wuhan, China), and ELISA Kit for Vascular Endothelial Growth Factor Receptor 2 (VEGFR2) (Cloud-Clone Corp., Wuhan, China). The intra-assay coefficients of variation (CVs) were 6.2%, <10%, and <10%, respectively. Inter-assay CVs were 4.8%, <12%, and <12%, respectively. An ‘ImmunomatTM’ automatic microplate immunoassay was used for the studies.

### 2.3. Statistical Analysis

The Shapiro–Wilk test was used to assess the normality of the data distribution. Continuous variables were described using median and interquartile ranges (IQR), categorical variables were described using frequency and percentages. Intergroup differences were studied using the nonparametric Kruskal–Wallis H test for independent groups, and the Friedman test; post-hoc tests were performed using Dunn’s and Nemenyi’s tests. Categorical variables were analyzed using Fisher’s exact test. Regression analysis of relationships with age of VEGF-A, VEGFR-1, and VEGFR-2 in the control group was performed in the presence of a potential confounding bias. All analyses were carried out using IBM SPSS Statistics for Windows, Version 26.0. Armonk, NY: IBM Corp. The differences were considered significant at *p* < 0.05.

### 2.4. Power Calculation

In a study by Xue L et al. [41] the authors reported a VEGF-A level of 197.6 ± 34.3 pg/mL in patients with ischemic stroke on day 7, compared with 118.0 ± 13.2 pg/mL in patients in the control group. Assuming a mean of 200 pg/mL and pooled standard deviation of 80 units in the main group, and a mean of 118 in control group, the study would require a sample size of 20 patients for each group, to achieve power of 90% and a level of significance of 5% (two sided), for detecting true difference in means between the test and the reference groups.

## 3. Results

One hundred and eight patients met the inclusion criteria, including 70 patients with ischemic strokes (atherothrombotic, cardioembolic, lacunar) and 38 patients with hemorrhagic strokes (subarachnoid hemorrhage, intracerebral hemorrhage). The patients’ characteristics are summarized in Table 1. The VEGFR-1 and VEGFR-2 levels were assessed simultaneously in 57 patients, with repeated measurements taken in different stroke phases (blood samples were drawn once in 32 patients and 2–3 times in 25 patients at different disease phases) and in 20 control subjects, while VEGF-A values were evaluated in a separate group of 51 patients and in an independent control group of 20 patients (Table 2).

### 3.1. VEGF-A, VEGFR-1, VEGFR-2 in Different Phases of Ischemic Stroke

**Figure 1:**
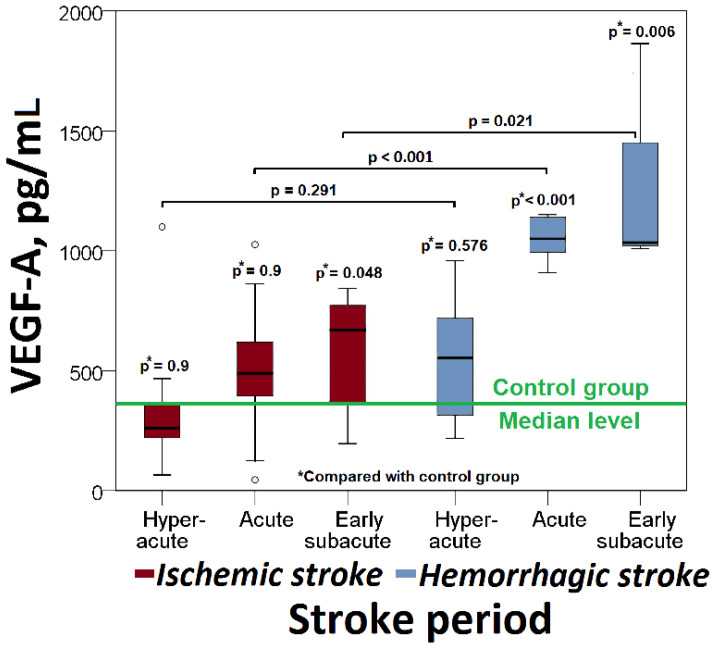
Changes in serum VEGF-A level in patients with ischemic or hemorrhagic stroke.

In ischemic stroke, there was a significant increase in VEGF-A levels in the early subacute phase (669 (IQR: 328–779) pg/mL vs. 355 (IQR: 133–406) pg/mL, *p* = 0.048) (Figure 1).

**Figure 2:**
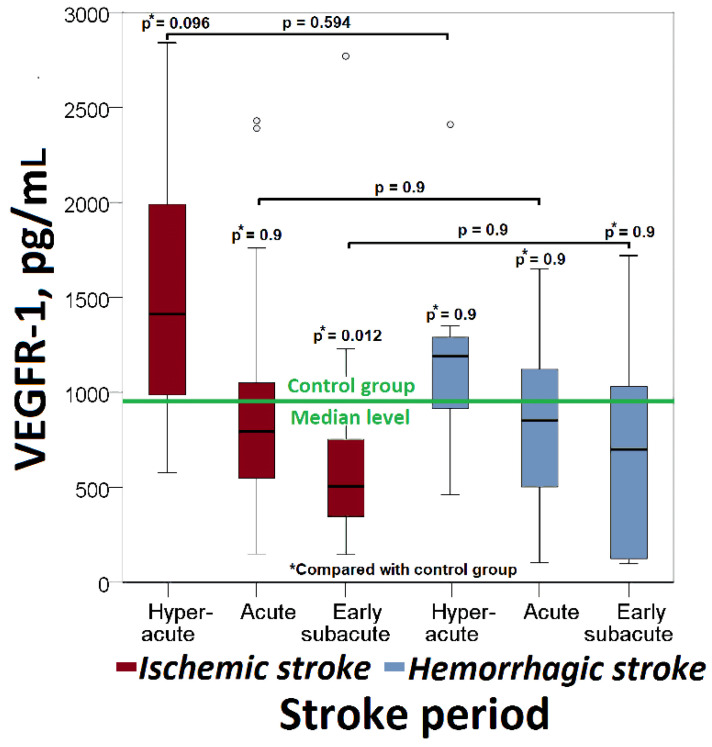
Changes in serum VEGFR-1 level in patients with ischemic or hemorrhagic stroke.

The median serum VEGFR-1 levels in patients with ischemic stroke in the hyper-acute phase were higher than the control group. In the acute and early subacute phases of ischemic stroke, median VEGFR-1 values were lower than the control values. The VEGFR-1 levels were significantly lower in the early subacute ischemic stroke group (505 (IQR: 345–753) pg/mL vs. 904 (IQR: 625–1118) pg/mL, *p* = 0.012), compared with controls (Figure 2).

**Figure 3:**
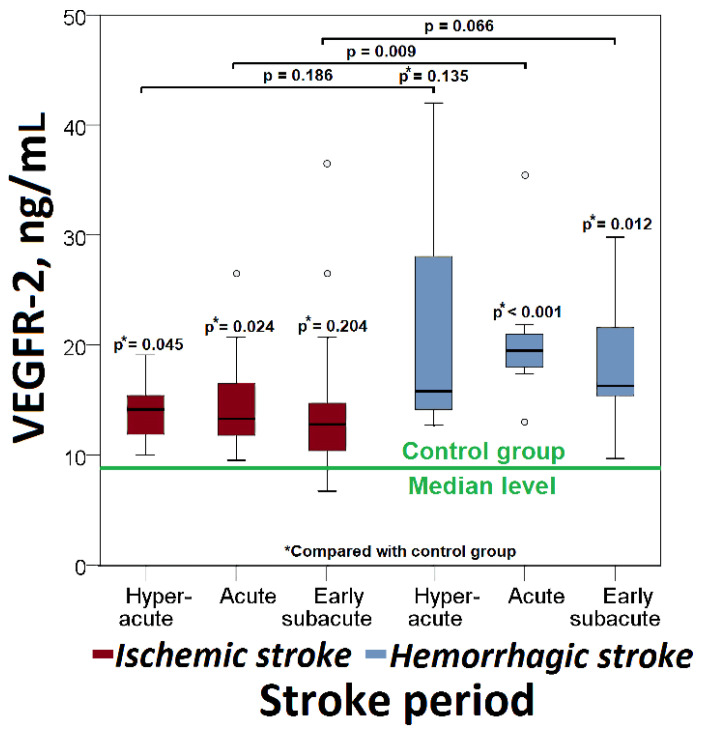
Changes in serum VEGFR-2 level in patients with ischemic or hemorrhagic stroke.

The median VEGFR-2 values in all phases of ischemic stroke were higher than the controls. The VEGFR-2 levels were significantly higher in the hyper-acute (14.2 (IQR: 11.9–15.6) ng/mL vs. 8.6 (IQR: 5.9–15.2) ng/mL, *p* = 0.045) and acute phases of ischemic stroke (13.3 (IQR: 11.7–16.6) ng/mL vs. 8.6 (IQR: 5.9–15.2) ng/mL, *p* = 0.024), compared with controls (Figure 3).

### 3.2. VEGF-A, VEGFR-1, VEGFR-2 in Different Phases of Hemorrhagic Stroke

VEGF-A levels increased significantly in the acute (1051 (IQR: 989–1146) pg/mL vs. 355 (IQR: 133–406) pg/mL, *p* < 0.001) and the early subacute phases (1034 (IQR: 1016–1657) pg/mL vs. 355 (IQR: 133–406) pg/mL, *p* = 0.006) of hemorrhagic stroke, compared with controls (Figure 1).

The median serum VEGFR-1 levels in patients with hyper-acute phase hemorrhagic strokes were higher than the controls. In the acute and early subacute phases of hemorrhagic stroke, median VEGFR-1 values were lower than the control values (Figure 2).

The median VEGFR-2 values in all phases of hemorrhagic stroke were higher than the controls. In hemorrhagic stroke, there was a significant increase in VEGFR-2 levels in the acute (19.5 (IQR: 17.4–21.9) ng/mL vs. 8.6 (IQR: 5.9–15.2) ng/mL, *p* < 0.001) and in the early subacute phases (16.3 (IQR: 15.1–23.1) ng/mL vs. 8.6 (IQR: 5.9–15.2) ng/mL, *p* = 0.012), compared with controls (Figure 3).

### 3.3. VEGF-A, VEGFR-1, VEGFR-2 in Different Types of Stroke

VEGF-A levels were significantly elevated in acute and early subacute hemorrhagic stroke compared with the corresponding phases of ischemic stroke (1051 (IQR: 989–1146) pg/mL vs. 489 (IQR: 260–742) pg/mL, *p* < 0.001), (1034 (IQR: 1016–1657) pg/mL vs. 669 (IQR: 328–779) pg/mL, *p* = 0.021), respectively) (Figure 1).

No significant differences in VEGFR-1 changes were found between hemorrhagic and ischemic strokes (*p* > 0.05, Figure 2).

VEGFR-2 levels increased more significantly in acute hemorrhagic stroke compared with the same phase of ischemic stroke (19.5 (IQR: 17.4–21.9) ng/mL vs. 13.3 (IQR: 11.7–16.6) ng/mL, *p* < 0.009) (Figure 3).

**Figure 4:**
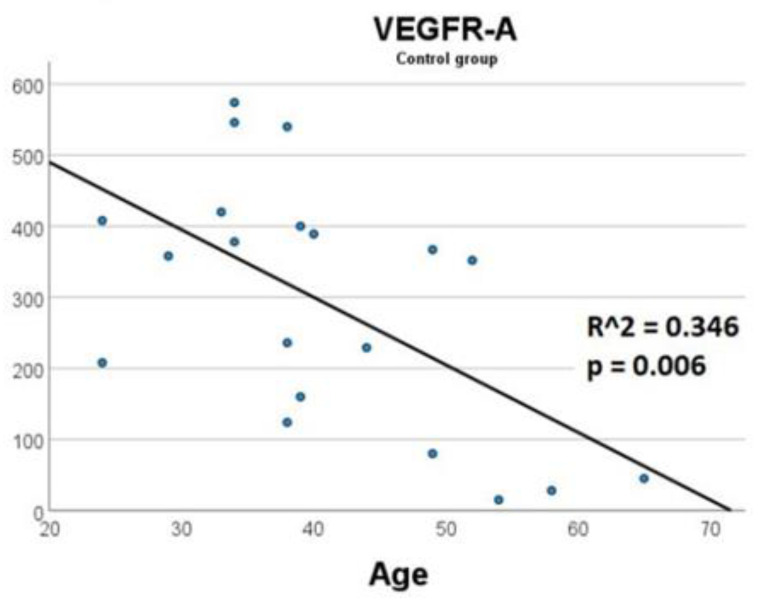
The relationship between the levels of VEGF-A and age in the control group.

To exclude the effect of age differences on the increase in soluble mediators in patients with stroke, we analyzed in the control group the relationship of VEGF-A, VEGFR-1, VEGFR-2 levels with age. No relationship between age and VEGFR-1 was found (R^2 = 0.001, *p* = 0.891), VEGFR-2 (R^2 = 0.016, *p* = 0.597). The relationship between age and VEGF-A was weakly negative (R^2 = 0.346, *p* = 0.006) (Figure 4). Therefore, the increases in circulating levels of VEGF-A and its receptors in the stroke group cannot be associated with age.

## 4. Discussion

Our results demonstrate different levels of VEGF-A and its receptors at various phases of ischemic and hemorrhagic stroke. In ischemic stroke, increased VEGFR-2 was found in the hyper-acute and acute phases, while elevated VEGF-A and reduced VEGFR-1 levels were revealed in the early subacute phase. In hemorrhagic stroke, no significant changes in levels of VEGF-A and its receptors were identified in the hyper-acute phase. In the acute and early subacute phases, however, there was an increase in levels of VEGF-A and VEGFR-2.

Although several studies have referred to VEGF in acute cerebrovascular accidents, its dynamics have been considered in only a few. Given the subtle and not fully understood mechanisms of the interaction of this factor with its receptors, studies of factor and receptor levels in various diseases should be carried out.

Previous studies noted increased VEGF levels in acute cerebrovascular accidents, in agreement with the results of our study [9,42]. Matsuo et al. showed that plasma VEGF values were significantly higher up to 90 days after stroke onset in all stroke subtypes compared with controls [43]. However, meta-analysis by Seidkhani-Nahal et al. showed that serum VEGF levels were not significantly associated with diagnosis of ischemic stroke. Therefore, the usefulness of VEGF as a stroke marker is questionable [44]. Meanwhile, Bhasin et al. pointed to the prognostic significance of VEGF indices in the acute stage of ischemic stroke [10].

The increase in VEGF-A levels in the early hemorrhagic stroke phase could indicate edema and the severity of tissue injury, which has been shown in a number of experimental studies [45].

Increased levels of circulating VEGF-A in the later phases of ischemic stroke are probably due to the activation of angiogenesis [33]. Alternatively, stroke-induced vascular damage in the blood–brain barrier leads to increased VEGF expression in the ischemic penumbra zone, from where it can enter the bloodstream when the blood–brain barrier is damaged [46,47].

We noted decreasing VEGFR-1 serum levels over time during acute and early subacute phases of ischemic and hemorrhagic strokes. The main functions of VEGFR-1 could be both the transmission of mitotic signals and downregulation of VEGF-A in vascular endothelial cells, supported by the existence of the soluble VEGR receptor (sVEGFR-1) [48]. This form is not a transmembrane protein; it lacks a tyrosine kinase domain and is unable to transmit the signal, which in turn leads to the suppression of angiogenesis. Assuming that VEGFR-1 is a “ligand trap” which binds excess VEGF-A in the blood of stroke patients, the reversed direction of changes in VEGFR-1 and VEGF-A levels can appear obvious [48,49]. A decrease in VEGFR-1 due to inhibition of protein synthesis also remains possible.

The level of VEGFR-2 rises earlier in ischemic stroke than in hemorrhagic stroke, while VEGF-A in ischemic stroke increases later than in hemorrhagic stroke. We found that VEGFR-2 increased in hyper-acute ischemic stroke, which could be considered a potential diagnostic marker. This is critically important because computed brain tomography, being the worldwide gold standard for the diagnosis of hemorrhagic stroke, can detect the ischemic zone in only one-third of cases of ischemic stroke [50].

It can be assumed that early elevation of blood VEGFR-2 levels in ischemic stroke could be due to hypoxia-induced increase of its expression in neurons and endothelial cells, and the subsequent increase in the blood-brain barrier permeability.

The elevation of VEGFR-2 in the hyper-acute and acute periods of ischemic stroke may be associated with the formation of the ischemic penumbra zone, as increased VEGFR-2 expression in the peri-infarct area was reported [51], as well as with microglia activation. In a morphological study of the brains of rats exposed to 100-min focal cerebral ischemia, the expression of VEGFR-2 was shown in activated microglia cells [52]. Because ischemic strokes are often atherothrombotic, the role of VEGFR-2 in the pathogenesis of atherosclerosis and atherosclerotic plaque lesions should be considered [53,54]. The dual functional role of VEGFR-2 should also be considered, because in addition to VEGFR-2 involvement in endothelial cell proliferation and inhibition of apoptosis, it can form a complex with adhesion molecules that weakens intercellular connections, alters the endothelial cytoskeleton, and induces endothelial fenestration, causing increased vascular permeability [30].

The VEGF/VEGFR signaling pathway has pleiotropic effects. On the one hand, its activation triggers brain angiogenesis associated with hypoxia and vascular damage, and has neuroprotective effects [55,56,57]. On the other hand, VEGF mediates the impairment of the blood–brain barrier and increased vascular permeability, which leads to edema and increased intracranial pressure [30,35,58].

Experimental studies are underway on the therapeutic efficacy of VEGF and anti-VEGF antibody neutralization for the treatment of stroke and its sequelae. However, the results of these studies are controversial. It has been revealed that anti-VEGF antibody can decrease blood–brain barrier (BBB) permeability, suppress brain edema formation, and improve functional outcome after subarachnoid hemorrhage [46]. It was demonstrated that inhibition of VEGF at the acute stage of stroke may reduce ischemic lesions, whereas delayed administration of VEGF during stroke recovery can markedly enhance angiogenesis in the ischemic brain and reduce neurological deficits [59,60]. Hence, timing of VEGF administration is crucial for its effects on ischemic brain tissue.

The dependence of the factor’s therapeutic effect on the time of administration may be due to the dynamics of BBB damage in acute cerebrovascular accidents.

It has been proposed that the process of BBB damage in ischemic stroke goes through several stages of development, depending on the stages of stroke. The first stage, corresponding to the hyper-acute stage of stroke, is characterized by increased BBB permeability and cytotoxic edema [61,62,63]. The second stage corresponds to the acute stage of stroke and is characterized by swelling of endothelial cells and astrocyte pedicles [61], leading to hypoperfusion. Hypoperfusion leads to increased damage to the BBB. At the third stage, corresponding to the subacute stage of stroke, reparative and regenerative processes take place, in particular, neoangiogenesis [64]. Experimental [63,65] and clinical [66] studies have shown that BBB permeability remains elevated for several weeks after a stroke, which can be explained by the immaturity of vessels, which are not sufficiently tight. At the chronic stage of stroke (>6 weeks), BBB permeability begins to decrease, which is associated with an increase in recovery factors [67].

Dysfunction of the BBB is an essential part of the pathophysiology of ischemic and hemorrhagic stroke. In ischemic stroke, BBB damage occurs due to abnormal paracellular and transcellular permeability and impaired endothelial structure. Abnormal water–electrolyte balance and leukocytic infiltration have also been observed. Two ultrastructural phases of BBB damage have been characterized. The first phase consists of changes in the endothelial cytoskeleton 30–60 min after ischemia, followed by enzymatic cleavage of dense contact proteins [68]. Abnormal pericyte function is an important component of the pathophysiology of BBB dysfunction [69]. Moreover, BBB damage further inhibits nervous recovery. Substantial evidence exists showing the effect of hypoxia and ischemia on the BBB, indicating the disruption of tight junctions and an increase of BBB permeability. These events appear to be mediated by the release of soluble factors, including cytokines, VEGF, and NO [70]. A murine study showed that VEGF initially contributed to the damage to the BBB, while in the post-stroke period it activated angiogenesis [71]. In SAH, the mechanism of BBB damage is similar to that in intracerebral hemorrhage and ischemic stroke, except for the initial period of the disease. Thus, in subarachnoid hemorrhage (SAH) the intracranial pressure increases due to blood leakage into the subarachnoid space, which subsequently reduces the microcirculatory blood flow [72]. Later, lack of ATP and increased levels of toxic metabolites in the blood initiate a pathological cascade including depolarization, excitotoxicity, cell edema, oxidative stress, and abnormal ion homeostasis, leading to secondary BBB damage. This can be defined as the secondary stage of BBB dysfunction [73,74]. Recovery of the BBB after its damage is a long process [75].

The results obtained in this study give us reason to assume that changes in serum levels of VEGF-A and its receptors in patients with hemorrhagic or ischemic stroke indicate different pathogenic pathways depending on the phase of the disease. In the hyper-acute and acute phases of ischemic or hemorrhagic stroke, an increase in VEGFR-2 levels indicates tissue change and circulatory disorders that predominate in early stroke. The increase in VEGF-A levels in the early subacute periods of hemorrhagic and ischemic strokes indicate ongoing regeneration, including neoangiogenesis.

Our study has some obvious limitations; we acknowledge the limits of its external validity. Another limitation is related to the retrospective design of the study. Furthermore, we did not analyze neurological and other clinical parameters. The above limitations might have biased our results.

Further studies are necessary to determine the patterns of changes in VEGF levels during various phases of stroke, and to unravel the relationship between these levels and stroke pathogenesis. An important line of research in future will be the precise assessment of serum levels of VEGF-A and its receptors for lacunar versus non-lacunar acute ischemic stroke, as the pathophysiology, prognosis, and clinical features of acute small-vessel ischemic strokes are different from other types of cerebral infarcts [76].

Full multivariate analysis should be performed in future studies, assessing the clinical significance of changes in levels of molecular markers, with a larger cohort of patients to allow validation of our findings with a more personalized patient approach. A multicenter randomized trial could provide a rationale for using serum VEGF-A and VEGFR-2 levels in clinical practice for differential diagnosis of types and phases of acute cerebrovascular incidents.

## 5. Conclusions

Our results demonstrate different levels of VEGF-A and its receptors at various phases of ischemic and hemorrhagic stroke. In ischemic stroke, increased VEGFR-2 levels were found in the hyper-acute and acute phases. Levels of VEGF-A were elevated in the acute and early subacute phases. In the early subacute phase, reduced VEGFR-1 levels were revealed.

In the acute and early subacute phases of hemorrhagic stroke, there was an increase in the levels of VEGF-A and VEGFR-2.

## Figures and Tables

**Table 1:** Baseline characteristics of the patient cohort in the study.

Parameters	Main Group	Control Group	*p*-Value
N	108	40	-
Sex (M)	58 (53.7%)	19 (47.5%)	0.6
Age (years)	68.0 (57.0–79.0), range: 25–89	52.5 (38.3–60.0), range: 24–68	<0.001 *
Types and phases of strokes			
Ischemic stroke N = 70 (64.8%)		Hemorrhagic stroke N = 38 (35.2%)	
Age (years):	75.0 (62.8–80.0)	58.5 (45.0–62.0)	<0.001 *
Sex (M):	35 (50.0%)	23 (60.5%)	0.4
Measurements, N Hyper-acute (N = 35) Acute (N = 42) Early subacute (N = 37)		Measurements, N Hyper-acute (N = 15) Acute (N = 15) Early subacute (N = 13)	

* Differences are statistically significant.

**Table 2:** Values of VEGFR-1, VEGFR-2, and VEGF-A in patient groups (according to stroke type and phase).

Stroke Phase	Stroke Type	Value	VEGFR-1 (pg/mL)	VEGFR-2 (ng/mL)	VEGF-A (pg/mL)
Hyper-acute	Hemorrhagic	N	8	8	7
		**Median (IQR)**	**1190 (769–1350)**	**15.8 (13.6–33.6)**	**554 (241–869)**
		Min	461	12,7	218
		Max	2410	42,0	959
	Ischemic	N	18	18	13
		**Median (IQR)**	**1412 (957–2055)**	**14,2 (11.9–15.6)**	**261 (220–410)**
		Min	577	10.0	65
		Max	2840	19.1	1101
Acute	Hemorrhagic	N	7	7	8
		**Median (IQR)**	**851 (409–1310)**	**19.5 (17.4–21.9)**	**1051 (989–1146)**
		Min	105	13.0	909
		Max	1650	35.4	2221
	Ischemic	N	33	33	9
		**Median (IQR)**	**793 (535–1055)**	**13.3 (11.7–16.6)**	**489 (260–742)**
		Min	149	9.5	45
		Max	3910	26.5	1026
Early subacute	Hemorrhagic	N	9	9	4
		**Median (IQR)**	**700 (118–1240)**	**16.3 (15.1–23.1)**	**1034 (1016–1657)**
		Min	99	9.7	1010
		Max	1720	29.8	1864
	Ischemic	N	27	27	10
		**Median (IQR)**	**505 (345–753)**	**12.8 (10.4–14.8)**	**669 (328–779)**
		Min	145	6.7	196
		Max	2770	36.5	844
Control	N/A	N	20	20	20
		**Median (IQR)**	**904 (625–1118)**	**8.6 (5.9–15.2)**	**355 (133–406)**
		Min	260	3.7	15
		Max	1650	19.6	574

N/A—not applicable.

## Data Availability

The data that support the findings of this study are available from the corresponding author upon reasonable request. Participant data without names and identifiers will be made available after approval from the corresponding author and local ethics committee.

## Supplementary Materials

The following supporting information can be downloaded at: https://www.mdpi.com/article/10.3390/cimb44100332/s1.

## References

[1] Mazilina A.N., Skalny A.V., Fesyun A.D., Yakovlev M.Y., Savko  S.A., Namiot  E.D. (2022). Review of the Elemental Status in Blood Serum in Patients with Ischemic Stroke. Bull. Rehabil. Med..

[2] Tokshilykova A., Kabdrakhmanova G., Sarkulova Z., Utepkaliyeva A., Khamidulla A., Urasheva Z. (2020). Modern Aspects of Etiopathogenesis, Diagnosis and Treatment of Hemorrhagic Stroke NcJSC.

[3] Wijerathne H., Witek M.A., Baird A.E., Soper S.A. (2020). Liquid biopsy markers for stroke diagnosis. Expert Rev. Mol. Diagn..

[4] Simpkins A.N., Janowski M., Oz H.S., Roberts J., Bix G., Doré S., Stowe A.M. (2020). Biomarker Application for Precision Medicine in Stroke. Transl. Stroke Res..

[5] Brunkhorst R., Pfeilschifter W., Foerch C. (2010). Astroglial proteins as diagnostic markers of acute intracerebral hemorrhage—Pathophysiological background and clinical findings. Transl. Stroke Res..

[6] Golubev A.M., Grechko A.V., Govorukhina M.A., Zakharchenko V.E., Kuzovlev A.N., Petrova M.V. (2020). Molecular Markers of Hemorrhagic Stroke. Gen. Reanimatol..

[7] Escudero C., Acurio J., López E., Rodríguez A., Benavente A., Lara E., Korzeniewski S.J. (2021). Vascular endothelial growth factor and poor prognosis after ischaemic stroke. Eur. J. Neurol..

[8] Okazaki H., Beppu H., Mizutani K., Okamoto S., Sonoda S. (2014). Changes in serum growth factors in stroke rehabilitation patients and their relation to hemiparesis improvement. J. Stroke Cerebrovasc. Dis..

[9] Alrafiah A., Alofi E., Almohaya Y., Hamami A., Qadah T., Almaghrabi S., Hakami N., Al-rawaili M.S., Tayeb H.O. (2021). Angiogenesis Biomarkers in Ischemic Stroke Patients. J. Inflamm. Res..

[10] Bhasin A., Srivastava M., Vivekanandhan S., Moganty R., Talwar T., Sharma S., Kuthiala N., Kumaran S., Bhatia R. (2019). Vascular Endothelial Growth Factor as Predictive Biomarker for Stroke Severity and Outcome: An Evaluation of a New Clinical Module in Acute Ischemic Stroke. Neurol. India.

[11] Wong Y.H., Wu C.C., Lai H.Y., Jheng B.R., Weng H.Y., Chang T.H., Chen B.S. (2015). Identification of network-based biomarkers of cardioembolic stroke using a systems biology approach with time series data. BMC Syst. Biol..

[12] Liu J., Li J. (2022). Astrocytes Protect Human Brain Microvascular Endothelial Cells from Hypoxia Injury by Regulating VEGF Expression. J. Healthc. Eng..

[13] Wang K., Lei L., Cao J., Qiao Y., Liang R., Duan J., Feng Z., Ding Y., Ma Y., Yang Z. (2021). Network pharmacology-based prediction of the active compounds and mechanism of Buyang Huanwu Decoction for ischemic stroke. Exp. Ther. Med..

[14] Jelkmann W. (2001). Pitfalls in the measurement of circulating vascular endothelial growth factor. Clin. Chem..

[15] da Silva T.M.V., Stein A.M., de Melo Coelho F.G., Rueda A.V., Camarini R., Galduróz R.F. (2022). Circulating levels of vascular endothelial growth factor in patients with Alzheimer’s disease: A case-control study. Behav. Brain Res..

[16] Belgore F.M., Blann A.D., Li-Saw-Hee F.L., Beevers D.G., Lip G.Y. (2001). Plasma levels of vascular en-dothelial growth factor and its soluble receptor (SFlt-1) in essential hypertension. Am. J. Cardiol..

[17] Blann A.D., Belgore F.M., McCollum C.N., Silverman S., Lip P.L., Lip G.Y. (2002). Vascular endothelial growth factor and its receptor, Flt-1, in the plasma of patients with coronary or peripheral atherosclerosis, or Type II diabetes. Clin. Sci..

[18] Hojo Y., Ikeda U., Zhu Y., Okada M., Ueno S., Arakawa H., Fujikawa H., Katsuki T., Shimada K. (2000). Expression of vascular endothelial growth factor in patients with acute myocardial infarction. J. Am. Coll. Cardiol..

[19] Chin B.S., Chung N.A., Gibbs C.R., Blann A.D., Lip G.Y. (2002). Vascular endothelial growth factor and soluble P-selectin in acute and chronic congestive heart failure. Am. J. Cardiol..

[20] Neufeld G., Cohen T., Gengrinovitch S., Poltorak Z. (1999). Vascular endothelial growth factor (VEGF) and its receptors. FASEB J..

[21] Peach C.J., Mignone V.W., Arruda M.A., Alcobia D.C., Hill S.J., Kilpatrick L.E., Woolard J. (2018). Molecular Pharmacology of VEGF-A Isoforms: Binding and Signalling at VEGFR2. Int. J. Mol. Sci..

[22] Marks E.C.A., Wilkinson T.M., Frampton C.M., Skelton L., Pilbrow A.P., Yandle T.G., Pemberton C.J., Doughty R.N., Whalley G.A., Ellis C.J. (2018). Plasma levels of soluble VEGF receptor isoforms, circulating pterins and VEGF system SNPs as prognostic biomarkers in patients with acute coronary syndromes. BMC Cardiovasc. Disord..

[23] Cárdenas-Rivera A., Campero-Romero A.N., Heras-Romero Y., Penagos-Puig A., Rincón-Heredia R., Tovar-Y-Romo L.B. (2019). Early Post-stroke Activation of Vascular Endothelial Growth Factor Receptor 2 Hinders the Receptor 1-Dependent Neuroprotection Afforded by the Endogenous Ligand. Front. Cell. Neurosci..

[24] Rosen L.S. (2002). Clinical experience with angiogenesis signaling inhibitors: Focus on vascular endothelial growth factor (VEGF) blockers. Cancer Control.

[25] Yamazaki Y., Morita T. (2006). Molecular and functional diversity of vascular endothelial growth factors. Mol. Divers..

[26] Takahashi H., Shibuya M. (2005). The vascular endothelial growth factor (VEGF)/VEGF receptor system and its role under physiological and pathological conditions. Clin. Sci..

[27] Duffy A.M., Bouchier-Hayes D.J., Harmey J.H. Vascular Endothelial Growth Factor (VEGF) and Its Role in Non-Endothelial Cells: Autocrine Signalling by VEGF. Madame Curie Bioscience Database. Austin, TX: Landes Bioscience, 2000–2013.

[28] Tjwa M., Luttun A., Autiero M., Carmeliet P. (2003). VEGF and PlGF: Two pleiotropic growth factors with distinct roles in development and homeostasis. Cell Tissue Res..

[29] Dragoni S., Turowski P. (2018). Polarised VEGFA Signalling at Vascular Blood–Neural Barriers. Int. J. Mol. Sci..

[30] Melincovici C.S., Boşca A.B., Şuşman S., Mărginean M., Mihu C., Istrate M., Moldovan I.M., Roman A.L., Mihu C.M. (2018). Vascular endothelial growth factor (VEGF)—Key factor in normal and pathological angiogenesis. Rom. J. Morphol. Embryol..

[31] Jean LeBlanc N., Guruswamy R., ElAli A. (2018). Vascular Endothelial Growth Factor Isoform-B Stimulates Neurovascular Repair After Ischemic Stroke by Promoting the Function of Pericytes via Vascular Endothelial Growth Factor Receptor-1. Mol. Neurobiol..

[32] Hao T., Rockwell P. (2013). Signaling through the vascular endothelial growth factor receptor VEGFR-2 protects hippocampal neurons from mitochondrial dysfunction and oxidative stress. Free Radic. Biol. Med..

[33] Li T., Zhu Y., Han L., Ren W., Liu H., Qin C. (2015). VEGFR-1 activation induced MMP-9-dependent invasion in hepatocellular carcinoma. Future Oncol..

[34] Wittko-Schneider I.M., Schneider F.T., Plate K.H. (2013). Brain homeostasis: VEGF receptor 1 and 2—Two unequal brothers in mind. Cell Mol. Life Sci..

[35] Geiseler S.J., Morland C. (2018). The Janus Face of VEGF in Stroke. Int. J. Mol. Sci..

[36] Greenberg D.A., Jin K. (2013). Vascular endothelial growth factors (VEGFs) and stroke. Cell Mol. Life Sci..

[37] Carmeliet P., Ruiz de Almodovar C. (2013). VEGF ligands and receptors: Implications in neurodevelopment and neurodegeneration. Cell. Mol. Life Sci..

[38] Schoch H.J., Fischer S., Marti H.H. (2002). Hypoxia-induced vascular endothelial growth factor expression causes vascular leakage in the brain. Brain.

[39] von Elm E., Altman D.G., Egger M., Pocock S.J., Gotzsche P.C., Vandenbroucke J.P. (2007). TROBE Initiative: The strengthening the reporting of observational studies in epidemiology (STROBE) statement: Guidelines for reporting observational studies. BMJ.

[40] Bernhardt J., Hayward K., Kwakkel G., Ward N., Wolf S.L., Borschmann K., Krakauer J.W., Boyd L.A., Carmichael S.T., Corbett D. (2017). Agreed definitions and a shared vision for new standards in stroke recovery research: The Stroke Recovery and Rehabilitation Roundtable taskforce. Int. J. Stroke.

[41] Xue L., Chen H., Zhang T., Chen J., Geng Z., Zhao Y. (2017). Changes in serum vascular endothelial growth factor and endostatin concentrations associated with circulating endothelial progenitor cells after acute ischemic stroke. Metab. Brain Dis..

[42] Prodjohardjono A., Vidyanti A.N., Susianti N.A., Sudarmanta, Sutarni S., Setyopranoto I. (2020). Higher level of acute serum VEGF and larger infarct volume are more frequently associated with post-stroke cognitive impairment. PLoS ONE.

[43] Matsuo R., Ago T., Kamouchi M., Kuroda J., Kuwashiro T., Hata J., Sugimori H., Fukuda K., Gotoh S., Makihara N. (2013). Clinical significance of plasma VEGF value in ischemic stroke—Research for biomarkers in ischemic stroke (REBIOS) study. BMC Neurol..

[44] Seidkhani-Nahal A., Khosravi A., Mirzaei A., Basati G., Abbasi M., Noori-Zadeh A. (2021). Serum vascular endothelial growth factor (VEGF) levels in ischemic stroke patients: A systematic review and meta-analysis of case–control studies. Neurol. Sci..

[45] Liu L., Fujimoto M., Kawakita F., Ichikawa N., Suzuki H. (2016). Vascular Endothelial Growth Factor in Brain Edema Formation After Subarachnoid Hemorrhage. Acta Neurochir Suppl..

[46] Åberg N.D., Wall A., Anger O., Jood K., Andreasson U., Blennow K., Zetterberg H., Isgaard J., Jern C., Svensson J. (2020). Circulating levels of vascular endothelial growth factor and post-stroke long-term functional outcome. Acta Neurol. Scand..

[47] Shibuya M. (2006). Differential roles of vascular endothelial growth factor receptor-1 and receptor-2 in angiogenesis. J. Biochem. Mol. Biol..

[48] Rud’ko A.S., Efendieva M.K., Budzinskaia M.V., Karpilova M.A. (2017). Influence of vascular endothelial growth factor on angiogenesis and neurogenesis. Vestn. Oftalmol..

[49] Stevens M., Oltean S. (2019). Modulation of Receptor Tyrosine Kinase Activity through Alternative Splicing of Ligands and Receptors in the VEGF-A/VEGFR Axis. Cells.

[50] Chalela J.A., Kidwell C.S., Nentwich L.M., Luby M., Butman J.A., Demchuk A.M., Hill M.D., Patronas N., Latour L., Warach S. (2007). Magnetic resonance imaging and computed tomography in emergency assessment of patients with suspected acute stroke: A prospective comparison. Lancet.

[51] Kim I.D., Cave J.W., Cho S. (2021). Aflibercept, a VEGF (Vascular Endothelial Growth Factor)-Trap, Reduces Vascular Permeability and Stroke-Induced Brain Swelling in Obese Mice. Stroke.

[52] Esposito E., Hayakawa K., Ahn B.J., Chan S.J., Xing C., Liang A.C., Kim K.-W., Arai K., Lo E.H. (2018). Effects of ischemic post-conditioning on neuronal VEGF regulation and microglial polarization in a rat model of focal cerebral ischemia. J. Neurochem..

[53] Mao Y., Liu X., Song Y., Zhai C., Zhang L. (2018). VEGF-A/VEGFR-2 and FGF-2/FGFR-1 but not PDGF-BB/PDGFR-β play important roles in promoting immature and inflammatory intraplaque angiogenesis. PLoS ONE.

[54] de Vries M.R., Parma L., Peters H.A.B., Schepers A., Hamming J.F., Jukema J.W., Goumans M.J.T.H., Guo L., Finn A.V., Virmani R. (2019). Blockade of vascular endothelial growth factor receptor 2 inhibits intraplaque haemorrhage by normalization of plaque neovessels. J. Intern. Med..

[55] Mackenzie F., Ruhrberg C. (2012). Diverse roles for VEGF-A in the nervous system. Development.

[56] Qiu S., Wu T., Wang P., Li J., Li Q., Du J. (2016). The Association between VEGFR Gene Polymorphisms and Stroke: A Meta-Analysis. PLoS ONE.

[57] Góra-Kupilas K., Jośko J. (2005). The neuroprotective function of vascular endothelial growth factor (VEGF). Folia Neuropathol..

[58] Shim J.W., Madsen J.R. (2018). VEGF Signaling in Neurological Disorders. Int. J. Mol. Sci..

[59] Zhang H.T., Zhang P., Gao Y., Li C.L., Wang H.J., Chen L.C., Feng Y., Li R.Y., Li Y.L., Jiang C.L. (2017). Early VEGF inhibition attenuates blood-brain barrier disruption in ischemic rat brains by regulating the expression of MMPs. Mol. Med. Rep..

[60] Chan S.J., Esposito E., Hayakawa K., Mandaville E., Smith R., Guo S., Niu W., Wong P.T., Cool S.M., Lo E.H. (2020). Vascular endothelial growth factor 165-binding heparan sulfate promotes functional recovery from cerebral ischemia. Stroke.

[61] Lin L., Wang X., Yu Z. (2016). Ischemia-reperfusion injury in the brain: Mechanisms and potential therapeutic strategies. Biochem. Pharmacol. Open Access.

[62] Pillai D.R., Dittmar M., Baldaranov D., Heidemann R., Henning E.C., Schuierer G., Bogdahn U., Schlachetzki F. (2009). Cerebral ischemia-reperfusion injury in rats—A 3 T MRI study on biphasic blood-brain barrier opening and the dynamics of edema formation. J. Cereb. Blood Flow. Metab..

[63] Durukan A., Marinkovic I., Strbian D., Pitkonen M., Pedrono E., Soinne L., Abo-Ramadan U., Tatlisumak T. (2009). Post-ischemic blood-brain barrier leakage in rats: One-week follow-up by MRI. Brain Res..

[64] Sargento-Freitas J., Aday S., Nunes C., Cordeiro M., Gouveia A., Silva F., Machado C., Rodrigues B., Santo G.C., Ferreira C. (2018). Endothelial progenitor cells enhance blood-brain barrier permeability in subacute stroke. Neurology.

[65] Abo-Ramadan U., Durukan A., Pitkonen M., Marinkovic I., Tatlisumak E., Pedrono E., Soinne L., Strbian D., Tatlisumak T. (2009). Post-ischemic leakiness of the blood-brain barrier: A quantitative and systematic assessment by Patlak plots. Exp. Neurol..

[66] Müller S., Kufner A., Dell’Orco A., Rackoll T., Mekle R., Piper S.K., Fiebach J.B., Villringer K., Flöel A., Endres M. (2021). Evolution of Blood-Brain Barrier Permeability in Subacute Ischemic Stroke and Associations With Serum Biomarkers and Functional Outcome. Front. Neurol..

[67] Bernardo-Castro S., Sousa J.A., Brás A., Cecília C., Rodrigues B., Almendra L., Sargento-Freitas J. (2020). Pathophysiology of blood–brain barrier permeability throughout the different stages of ischemic stroke and its implication on hemorrhagic transformation and recovery. Front. Neurol..

[68] Sweeney M.D., Zhao Z., Montagne A., Nelson A.R., Zlokovic B.V. (2019). Blood-Brain Barrier: From Physiology to Disease and Back. Physiol. Rev..

[69] Bertrand L., Cho H.J., Toborek M. (2019). Blood–brain barrier pericytes as a target for HIV-1 infection. Brain.

[70] Kadry H., Noorani B., Cucullo L. (2020). A blood–brain barrier overview on structure, function, impairment, and biomarkers of integrity. Fluids Barriers CNS.

[71] Kadry H., Noorani B., Cucullo L. (2021). Blood-Brain Barrier Dysfunction in CNS Disorders and Putative Therapeutic Targets: An Overview. Pharmaceutics.

[72] Solár P., Zamani A., Lakatosová K., Joukal M. (2022). The blood–brain barrier and the neurovascular unit in subarachnoid hemorrhage: Molecular events and potential treatments. Fluids Barriers CNS.

[73] Topkoru B., Egemen E., Solaroglu I., Zhang J.H. (2017). Early brain injury or vasospasm? An overview of common mechanisms. Expert Rev. Cardiovasc. Ther..

[74] Fang Y., Gao S., Wang X., Cao Y., Lu J., Chen S., Lenahan C., Zhang J.H., Shao A., Zhang J. (2020). Programmed Cell Deaths and Potential Crosstalk With Blood-Brain Barrier Dysfunction After Hemorrhagic Stroke. Front. Cell. Neurosci..

[75] Jiang X., Andjelkovic A.V., Zhu L., Yang T., Bennett M.V.L., Chen J., Keep R.F., Shi Y. (2018). Blood-brain barrier dysfunction and recovery after ischemic stroke. Prog. Neurobiol..

[76] Rudilosso S., Rodríguez-Vázquez A., Urra X., Arboix A. (2022). The Potential Impact of Neuroimaging and Translational Research on the Clinical Management of Lacunar Stroke. Int. J. Mol. Sci..

